# Phase II Trial of Hypofractionated Radiotherapy and Immunochemotherapy in Primary Refractory Diffuse Large B‐Cell Lymphoma: Preliminary Results and Insights from Digital Spatial Profiling

**DOI:** 10.1002/mco2.70225

**Published:** 2025-05-25

**Authors:** Yong Yang, Jing Yu, Xiao‐Mei Hu, Si‐Lin Chen, Rui‐Zhi Zhao, Cheng Huang, Jiang‐Rui Guo, Tian‐Lan Tang, Cheng Chen, Yu‐Ping Lin, Ying Wang, Tian‐Xiu Liu, Hao Zheng, Si‐Qin Liao, Jin‐Hua Chen, Hai‐Ying Fu, Ting‐Bo Liu

**Affiliations:** ^1^ Department of Radiation Oncology Fujian Medical University Union Hospital, Fujian Key Laboratory of Intelligent Imaging and Precision Radiotherapy for Tumors (Fujian Medical University), Clinical Research Center for Radiology and Radiotherapy of Fujian Province (Digestive, Hematological and Breast Malignancies) Fuzhou China; ^2^ Department of Pulmonary Oncology Hubei Key Laboratory of Tumor Biological Behaviors, Hubei Cancer Clinical Study Center, Zhongnan Hospital of Wuhan University Wuhan China; ^3^ Department of Pathology Fujian Medical University Union Hospital Fuzhou China; ^4^ Department of Hematology Fujian Medical University Union Hospital, Fujian Institute of Hematology, Fujian Provincial Key Laboratory on Hematology Fuzhou China; ^5^ Department of PET/CT Fujian Medical University Union Hospital Fuzhou China; ^6^ Follow‐Up Center Fujian Medical University Union Hospital Fuzhou China; ^7^ Department of Hematology The Third Affiliated People's Hospital of Fujian University of Traditional Chinese Medicine, The Third People's Hospital of Fujian Province Fuzhou China

**Keywords:** diffuse large B‐cell lymphoma, immune system, primary refractory, radiotherapy, zimberelimab

## Abstract

This open‐label, single‐arm phase II study assessed the safety and efficacy of sequential hypofractionated radiotherapy (RT) followed by zimberelimab and R‐GemOx (rituximab, gemcitabine, oxaliplatin) in patients with primary refractory diffuse large B‐cell lymphoma (DLBCL). Fourteen patients were enrolled between June 2022 and December 2023, with 13 included in the analysis. RT doses of 36 and 24 Gy were delivered to the gross and target volumes in 12 fractions, followed by zimberelimab and R‐GemOx. The overall response rate within the irradiated field was 92.3%, and a complete response (CR) was achieved by 61.5% of patients; however, 38.5% experienced disease progression. Treatment‐related toxicities were manageable, primarily comprising mild leukocytopenia. Digital spatial profiling revealed 53 differentially expressed genes in CD20‐rich lymphoma regions and 93 in CD3‐rich T cell regions in non‐CR patients. Reactome analysis identified key immune system pathways. T cell infiltration correlated with treatment efficacy, and multiplex immunohistochemistry validated immune pathways as potential therapeutic targets. This study demonstrated the promising role of RT combined with immunochemotherapy in refractory DLBCL and suggests immune pathways as critical targets to improve treatment outcomes.

## Graphical abstract

This prospective study evaluates hypofractionated radiotherapy combined with zimberelimab in primary refractory DLBCL. A 61.5% complete response rate was achieved in the first stage. Digital spatial profiling analysis revealed T cell–lymphoma interactions and linked T cell infiltration to resistance. The findings highlight the role of the immune microenvironment and warrant further validation in preclinical models.

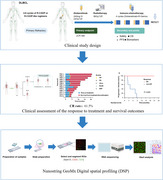

## 1 Background

The most commonly encountered lymphoid malignancy is diffuse large B‐cell lymphoma (DLBCL), accounting for 32% of the annual non‐Hodgkin lymphoma diagnoses [1, 2]. The R‐CHOP regimen, comprising rituximab, cyclophosphamide, doxorubicin, vincristine, and prednisone, has been widely recognized for its efficacy in improving survival outcomes and achieving remission in more than half of DLBCL cases [3, 4, 5]. Despite first‐line treatment, nearly 25% of patients fail to achieve a complete response (CR) and are categorized as having primary refractory disease [6, 7]. Second‐line therapy for these patients usually involves salvage chemotherapy followed by autologous stem cell transplantation (ASCT) or chimeric antigen receptor T‐cell (CAR‐T cell) therapy [8, 9, 10, 11, 12].

Radiotherapy (RT) serves as an effective local strategy to diminish the residual tumor burden before transplantation or CAR‐T cell therapy, potentially enhancing the cure rate for a subset of patients [13, 14]. Beyond local tumor reduction, hypofractionated bridge RT might also enhance the systemic efficacy of cellular immunotherapy, such as CAR‐T cell therapy, by promoting the release of tumor antigens and altering the tumor immune microenvironment (TIME) [15, 16]. A key mechanism by which RT affects the TIME is its ability to enhance T cell infiltration and activation within the tumor microenvironment (TME) [17]. RT has been found to upregulate the expression of specific chemokines, including C–C motif chemokine ligand 5 (CCL5) and C–X–C motif chemokine ligand 9 (CXCL9), which play crucial roles in recruiting and guiding T cells to the tumor site, further improving T‐cell‐mediated tumor control [18, 19].

Inspired by these findings, combining RT with immune checkpoint inhibitors (ICIs) presents a promising research avenue to treat DLBCL. Despite the demonstrated safety and efficacy of hypofractionated RT, out‐of‐field failure remains a significant barrier to its widespread acceptance [20, 21]. Therefore, identifying a gene signature associated with treatment efficacy and failure patterns could facilitate the identification of patients who are most likely to benefit from hypofractionated RT [22, 23]. Leveraging technological advancements in spatial biology, digital spatial profiling (DSP) offers a powerful tool to complement these clinical findings by enabling high‐resolution, spatially resolved analysis of gene expression within the TME [24]. Unlike bulk RNA sequencing, which provides an average gene expression across a tissue sample, DSP enables gene expression mapping at the cellular level, distinguishing immune and tumor cell populations in distinct tumor regions. In this study, we focused on lymphoma and T cell regions. By identifying differentially expressed genes (DEGs) in these areas, DSP can reveal potential biomarkers linked to treatment efficacy or failure, thereby refining treatment strategies for patients with refractory disease.

Based on our experience with hypofractionated RT for DLBCL [20], we hypothesized that combining hypofractionated RT with immuno‐chemotherapy could be an effective treatment for primary refractory DLBCL. This study presents preliminary findings from our prospective phase II trial, which investigated molecular biomarkers associated with treatment efficacy.

## 2 Results

### 2.1 Patient Characteristics

**FIGURE 1:**
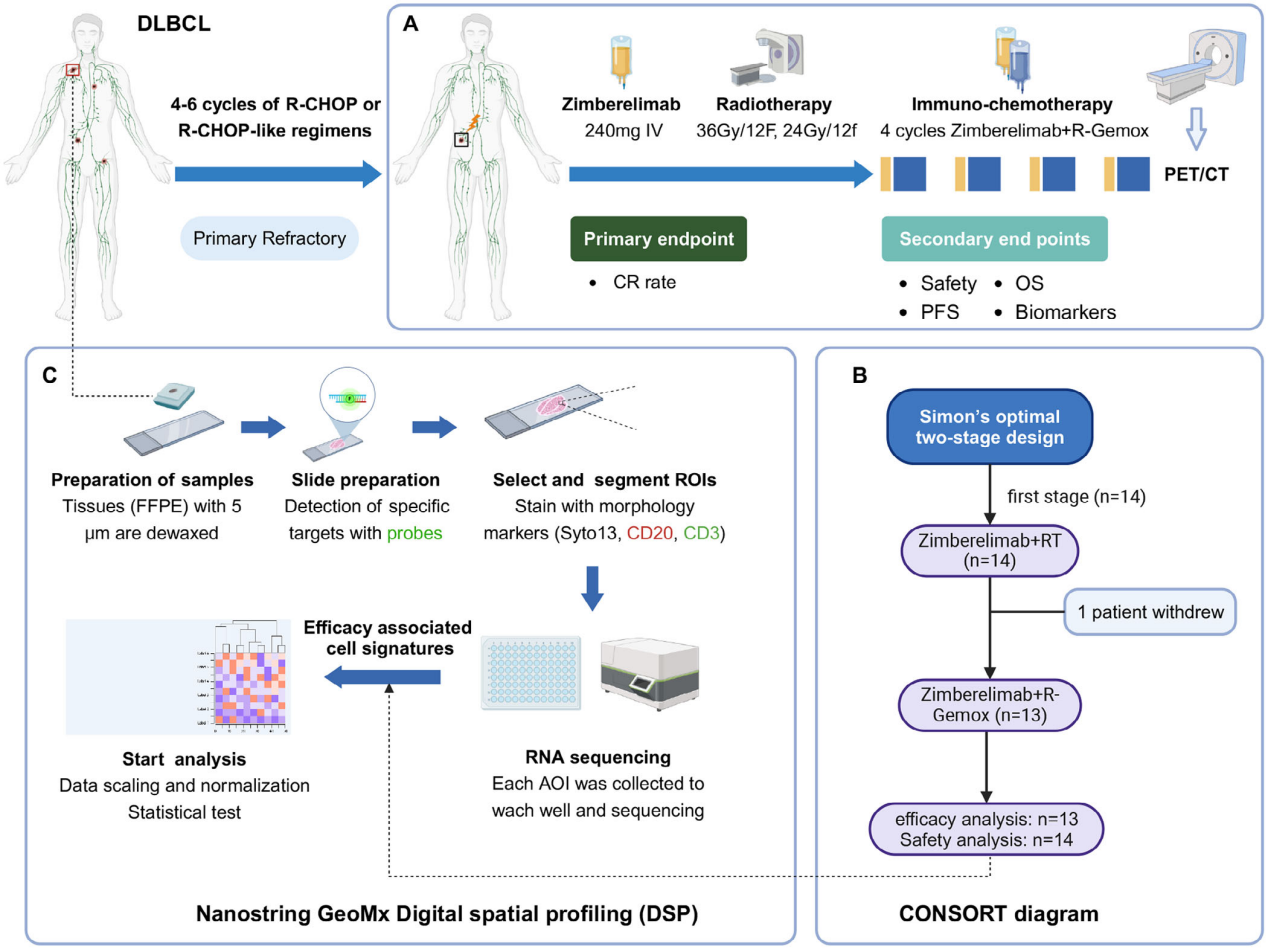
Study consort diagram. (A) Treatment and assessment; (B) study design and analysis of the population; (C) DSP examination. AOI, area of interest; CR, complete response; DLBCL, diffuse large B‐cell lymphoma; DSP, digital spatial profiling; FFPE, formalin‐fixed/paraffin‐embedded; R‐CHOP, rituximab with cyclophosphamide, doxorubicin, vincristine, and prednisone; R‐GemOx, rituximab with gemcitabine, and oxaliplatin; ROI, region of interest.

A total of 14 patients with primary refractory DLBCL were enrolled in the first stage of this study (between June 30, 2022 and November 30, 2023) (Figure 1B). The main clinical characteristics of the patients at initial diagnosis are provided in Table 1. The median age was 60 years (range, 19–75 years), with 55% of the patients being male, and all had an Eastern Cooperative Oncology Group (ECOG) performance status of 0–1. Most patients had extranodal involvement (85.7%) and advanced‐stage disease (57.2%), while 28.6% presented with B symptoms. In this cohort, nine patients (64.3%) were classified as the nongerminal center B‐cell‐like (non‐GCB) subtype according to the Hans method, and 42.9% had international prognostic index (IPI) scores ranging from 3 to 5. All enrolled patients received R‐CHOP or R‐CHOP‐like regimens as first‐line treatment, with most patients undergoing four cycles of chemotherapy. At the time of enrollment, 13 patients had achieved a partial response (PR), and one had progressive disease (PD).

**TABLE 1:** Clinical characteristics of the 13 enrolled patients with primary refractory DLBCL.

Patient	Sex	Age (years)	Initial staging	IPI scores	Cell of origin	Double hit	First‐line regimens and cycles	Response to first‐line therapy	Residual sites
#1	Female	19	IVB	2	Non‐GCB	No	RCEOP × 4	PR	Mediastinal
#2	Female	59	II	2	Non‐GCB	No	DA‐EPOCH‐R × 4	PR	Stomach
#3	Male	75	II	1	GCB	No	R‐CHOP × 6	PR	Colon
#4	Female	67	III	3	Non‐GCB	Yes	R‐CEOP × 4	PR	Colon
#5	Male	43	IVB	3	Non‐GCB	No	R‐CEOP × 6	PD	Stomach, para‐aortic
#6	Female	60	IV	3	GCB	No	R‐CHOP × 4	PR	Inguinal
#7	Male	63	IV	3	Non‐GCB	No	R‐CHOP × 4	PR	Submandibular
#8	Male	53	IVB	3	Non‐GCB	No	R‐CEOP × 4	PR	Left supraclavicular, axillary, para‐aortic, mesenteric
#9	Male	65	III	3	Non‐GCB	No	R‐CEOP × 4	PR	Left oropharynx
#10	Female	64	I	1	Non‐GCB	No	R‐CHOP × 6	PR	Left breast
#11	Female	68	II	2	Non‐GCB	No	R‐mini‐CEOP × 4	PR	Para‐aortic, right iliac, inguinal
#12	Male	50	I	1	GCB	No	R‐CEOP × 4	PR	Right maxillary sinus
#13	Male	39	IV	2	GCB	No	R‐CEOP × 4	PR	Para‐aortic

Abbreviations: DA‐EPOCH‐R, etoposide, prednisone, vincristine, cyclophosphamide, doxorubicin, rituximab; DLBCL, diffuse large B‐cell lymphoma; GCB, germinal center B‐cell; IPI, international prognostic index; PD, progressive disease; PR, partial response; R‐CEOP, rituximab, cyclophosphamide, etoposide, vincristine, prednisone; R‐CHOP, rituximab, cyclophosphamide, doxorubicin, vincristine, prednisone.

All patients received one dose of zimberelimab followed by intensity‐modulated RT or volumetric modulated arc therapy. The median volumes for gross tumor volume (GTV) and clinical target volume (CTV) were 28 mL (range, 2–483 mL) and 461 mL (range, 75–1084 mL), respectively. One patient withdrew from the study after completing RT, resulting in 13 patients being included in the intention‐to‐treat primary efficacy analysis. Of these 13 patients, 10 (76.9%) had a single residual lesion (oligoresidual disease) before RT. Following RT, 10 patients (76.9%) completed all four planned cycles of zimberelimab plus R‐GemOx. One patient experienced PD before the second dose of zimberelimab, another experienced PD after two cycles of R‐GemOx plus zimberelimab, and one patient refused further R‐GemOx plus zimberelimab after the third cycle.

### 2.2 Efficacy

**FIGURE 2:**
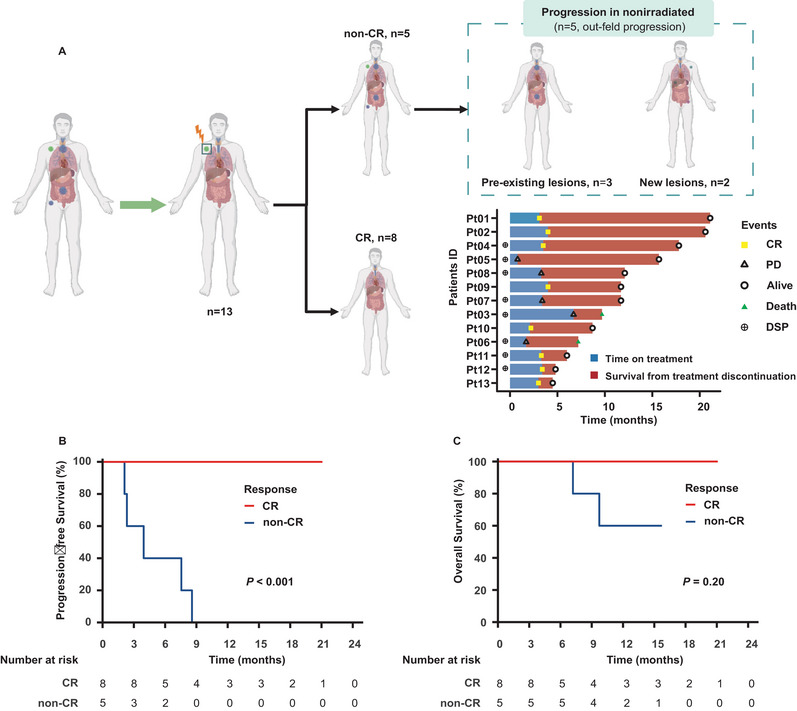
Clinical assessment of the response to treatment and survival outcomes for patients in the ITT population (*n* = 13). (A) The CR rate and failure patterns; (B) progression‐free survival; (C) overall survival. CR, complete response; DSP, digital spatial profiling; ITT, intention‐to‐treat; PD, progressive disease.

In the efficacy analysis set, all patients achieved a confirmed objective response, with eight achieving CRs (61.5%) and five experiencing PD (38.5%) (Table 2). The overall response rate (ORR (CR + PR)) was 57.1% (eight out of 14 patients). Additionally, within the irradiated field, the planning target volume (PTV) area ORR was 92.3% (12 out of 13), with 76.9% (10 out of 13) achieving CR. Only one patient developed progression within the RT field. Out‐of‐field progression was the most common first pattern of failure, occurring in all five patients who experienced progression (five out of five). This included three patients who failed to achieve CR after RT and two patients who initially achieved CR (Figure 2A). Compared with baseline positron emission tomography/computed tomography (PET/CT) imaging, among the five patients who experienced out‐of‐field progression after irradiation, three were categorized into the pre‐existing lesions group, and two into the new lesions group.

**TABLE 2:** Treatment and clinical outcomes of 13 enrolled patients.

Patient	First dose zimberelimab	RT dose	Second‐line regimens and cycles	CR within radiation field	CR after salvage therapy	Out‐field progression	Progression with new lesions	DSP	mIHC
#1	Yes	GTV 36 Gy, CTV 24Gy	R‐GemOx + zimberelimab × 4	Yes	Yes	No	No	NA	NA
#2	Yes	GTV 36 Gy, CTV 24Gy	R‐GemOx + zimberelimab × 4	Yes	Yes	No	No	NA	NA
#3	Yes	GTV 36 Gy, CTV 24Gy	R‐GemOx + zimberelimab × 4	Yes	No	Yes	No	Yes	Yes
#4	Yes	GTV 36 Gy, CTV 24Gy	R‐GemOx + zimberelimab × 4	Yes	Yes	No	No	Yes	Yes
#5	Yes	GTV 36 Gy, CTV 24Gy	R‐GemOx + zimberelimab × 1	No	No	Yes	Yes	Yes	NA
#6	Yes	GTV 36 Gy, CTV 24Gy	R‐GemOx + zimberelimab × 2	No	No	Yes	Yes	Yes	Yes
#7	Yes	GTV 36 Gy, CTV 24Gy	R‐GemOx + zimberelimab × 4	Yes	No	Yes	Yes	Yes	Yes
#8	Yes	GTV 36 Gy, CTV 24Gy	R‐GemOx + zimberelimab × 3	No	No	Yes	No	Yes	NA
#9	Yes	GTV 36 Gy, CTV 24Gy	R‐GemOx + zimberelimab × 4	Yes	Yes	No	No	NA	NA
#10	Yes	GTV 36 Gy, CTV 24Gy	R‐GemOx + zimberelimab × 3	Yes	Yes	No	No	NA	NA
#11	Yes	GTV 36 Gy, CTV 24Gy	R‐GemOx + zimberelimab × 3	Yes	Yes	No	No	Yes	Yes
#12	Yes	GTV 36 Gy, CTV 24Gy	R‐GemOx + zimberelimab × 4	Yes	Yes	No	No	Yes	Yes
#13	Yes	GTV 36 Gy, CTV 24Gy	R‐GemOx + zimberelimab × 4	Yes	Yes	No	No	NA	NA

Abbreviations: CR, complete response; CTV, clinical target volume; DSP, digital spatial profiling; GTV, gross target volume; NA, not applicable; R‐GemOx, rituximab, gemcitabine, oxaliplatin; RT, radiotherapy.

Following a median follow‐up of 11.7 months (range, 3.8–21.1 months), the median overall survival (OS) and progression‐free survival (PFS) were not reached. The 12‐month OS was 78.7% (95% CI: 56.4–100), and PFS was 54.9% (95% CI: 31.5–95.7). Patients who achieved a CR had a significantly better 12‐month PFS rate compared with those who did not (100 vs. 0%; *p* < 0.001; Figure 2B). The 12‐month OS showed no significant difference between the two groups (100 vs. 60%; *p* = 0.157; Figure 2C).

### 2.3 Safety

All patients experienced at least one treatment‐related adverse event (TRAE) following the initial dose of zimberelimab and RT, with the most frequent TRAEs being leukocytopenia (46.5%), nausea (42.9%), and oral mucositis (35.7%). Most TRAEs were grade 1–2, and only three patients experienced a grade 3 TRAE (leukocytopenia). No grade 4–5 TRAEs were reported (Table 3). During the subsequent period of zimberelimab with R‐GemOx, leukocytopenia remained the most common TRAE, and only three patients experienced grade 3 leukocytopenia. No patient‐reported serious AEs occurred, and no TRAE led to treatment discontinuation throughout the protocol therapy.

**TABLE 3:** Treatment‐related adverse events with protocol therapy.

Treatment‐related adverse events	No. (%)
Toxicities during first dose of zimberelimab (*n* = 14)	
Leukocytopenia	
Grade 1	2 (14.3)
Toxicities during RT (*n* = 14)	
Leukocytopenia	
Grade 1–2	9 (64.3)
Grade 3	2 (14.3)
Grade 4	1 (7.1)
Oral mucositis	
Grade 2	1 (7.1)
Gastrointestinal toxicity	
Grade 2	3 (21.4)
Toxicities during zimberelimab + R‐GemOx (*n* = 13)	
Leukocytopenia	
Grade 1–2	8 (61.5)
Grade 3	3 (23.1)
Thrombocytopenia	
Grade 1	1 (7.7)

Abbreviations: R‐GemOx, rituximab, gemcitabine, oxaliplatin; RT, radiotherapy.

### 2.4 Efficacy‐Associated Cell Signatures from DSP

**FIGURE 3:**
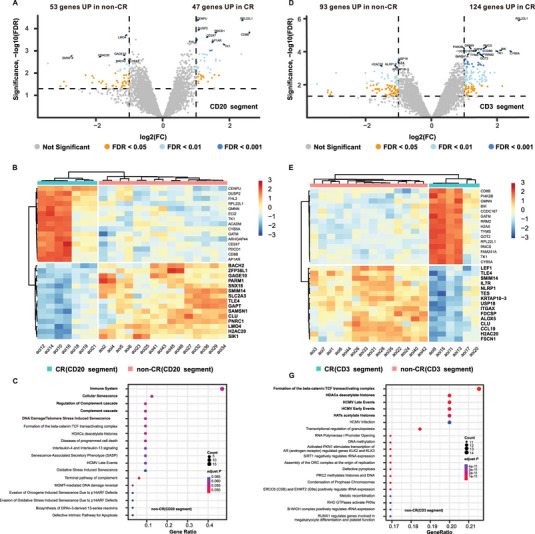
Digital spatial profiling analysis of lymphoma and T cells. (A) Volcano plots showing DEGs between lymphoma cells from CR and non‐CR patients; (B) heatmap displaying the top 15 highly expressed DEGs in lymphoma cells from CR and non‐CR patients; (C) Reactome pathway enrichment analysis of genes upregulated in non‐CR patients (CD20‐rich lymphoma); (D) volcano plots showing DEGs in T cells between CR and non‐CR patients; (E) heatmap displaying the top 15 highly expressed DEGs in T cells from CR and non‐CR patients; (F) Reactome pathway enrichment analysis of genes upregulated in non‐CR patients (CD3‐rich T cell AOIs). AOIs, areas of interest; CR, complete response; DEG, differentially expressed gene; FC, fold change; FDR, false discovery rate.

To experimentally investigate the actual inter‐lesional heterogeneity in the tumor cells and immune microenvironment, we selected eight nodal DLBCL cases (three with GCB, five with non‐GCB; Table 2). Analysis of DEGs in CD20‐rich lymphoma areas of interest (AOIs), identified 53 and 47 genes that were differentially upregulated between CD20‐rich and CD3‐rich AOIs in the non‐CR and CR patients, respectively (false discovery rate [FDR] < 0.05; Figure 3A), suggesting highly distinct gene expression patterns. Similarly, DEGs analysis in CD3‐rich T cell AOIs revealed that 93 and 124 genes were differentially upregulated in non‐CR and CR patients, respectively (FDR < 0.05; Figure 3D). The heatmaps displaying the top 15 highly expressed DEGs in CD20‐rich and CD3‐rich AOIs for non‐CR and CR patients are shown in Figure 3B,E, respectively.

For non‐CR patients, pathways related to the immune system and cellular senescence, such as those involving solute carrier family 2 member 3, interleukin 7 receptor, class II, DQ alpha 1 (HLA‐DQA1), major histocompatibility complex, cyclin dependent kinase inhibitor 2A, and H2A clustered histone 20, were enriched in CD20‐rich regions of interest (ROIs) (adjusted *p* < 0.001; Figure 3C). Similarly, for non‐CR patients, immune system pathways involving FYN binding protein, protein tyrosine phosphatase nonreceptor type 6, CD3 delta subunit of T‐cell receptor complex, suppressor of cytokine signaling 3, granzyme M, and T cell receptor‐associated transmembrane adaptor 1 were enriched in CD3‐rich ROIs (adjusted *p* < 0.001; Figure 3G). Interestingly, based on the DEGs in CD20‐rich and CD3‐rich AOIs, our spatial signaling analysis predicted a potential association between the ligands CCL19 and CCL21 with their receptor CCR7 on T cells. The remaining predicted ligand–receptor interactions can be found in Table S1.

### 2.5 T Cell Infiltration Associated with Efficacy

**FIGURE 4:**
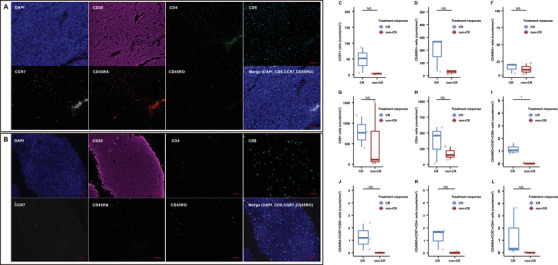
Representative mIHC images and quantification of T cell subsets in the tumor immune microenvironment of CR and non‐CR patients. (A and B) Representative mIHC images showing CD4, CD8, CCR7, CD45RA, and CD45RO expression level in the tumor immune microenvironment from patients achieving CR (A, *n* = 1) and non‐CR (B, *n* = 1). Scale bars: 100 µm. (C–L) Quantification of T cell infiltration levels in lymphoma tissues from patients achieving CR (*n* = 3) and non‐CR (*n* = 3), assessed using mIHC. The box plots illustrate immune cell densities, with the central line representing the median value, the box edges indicating the 25th and 75th percentiles, and the whiskers representing the minimum and maximum values. Statistical comparisons were conducted using a two‐tailed Student's *t*‐test. NS, not significant (*p* > 0.05); *, *p* ≤ 0.05. CCR7, C–C motif chemokine receptor 7; CD4, CD4 molecule; CD45RA, CD45 antigen long form; CD45RO, CD45 antigen short form; CD8, CD8 molecule; CR, complete response; mIHC, multiplex immunohistochemistry.

Tumor biopsies from six patients were available for multiplex immunohistochemistry (mIHC) analysis to validate the association between treatment efficacy and T cell infiltration identified by DSP. Representative mIHC images showed higher infiltration of immune cells in one patient who achieved CR compared with that in one patient did not achieve CR (Figure 4). There was a trend toward increased densities of CCR7+, CD45RO+, CD4+ T cells, and CD8+ T cells in the CR group (Figure S2). Patients with CR exhibited significantly greater densities of CCR7+CD45RO+CD8+ T cells (*p* = 0.009) compared with patients who did not achieve CR. Additionally, based on the results from the GSE31312 dataset, we further corroborated the association between T cell infiltration and treatment efficacy in DLBCL. The analysis revealed that higher CD4+ T cell abundance was associated with a trend toward better PFS (*p* = 0.06). Furthermore, higher CD8+ T cell abundance showed a significant association with improved PFS (*p* = 0.01). These findings provide further evidence that T cell infiltration, particularly CD4+ and CD8+ T cells, might be essential to improve treatment outcomes and prognosis in patients with DLBCL (Figure S1).

## 3 Discussion

This study is the first prospective investigation of hypofractionated RT combined with zimberelimab in patients with primary refractory DLBCL. Employing Simon's optimal two‐stage design, the primary endpoint was achieved by eight patients (61.5%) in the first stage, permitting the continuation of the study because of adequate CR rates. Our results indicated that hypofractionated RT achieved high local control rates, and the combination of zimberelimab and RT had a comparably safety profile to zimberelimab alone. Further exploratory analysis revealed spatial transcriptional diversity in regions segmented into T cells and lymphomas, highlighting their associations with clinical outcomes. We demonstrated that immune system pathway manipulation, particularly T cell infiltration, is associated with resistance to hypofractionated RT combined with immunotherapy in primary refractory DLBCL. Understanding these associations and validating the findings with cell culture and animal models will help to reveal the mechanisms underlying the synergistic effects of hypofractionated RT and immunotherapy in DLBCL.

Over the past two decades, the cure rate for first‐line DLBCL treatment has improved significantly. This enhancement is primarily attributed to incorporating rituximab (R) into the cyclophosphamide, doxorubicin, vincristine, and prednisolone (CHOP) chemotherapy regimen, along with an improved comprehension of the disease's heterogeneity and patient‐specific molecular profiles [3–5, 25]. Adding consolidation RT to the treatment regimen has notably reduced the risk of local failure in patients with a high recurrence risk, especially those with bulky disease [26, 27, 28, 29, 30, 31]. However, the critical question remains whether the risk of widespread failure outweighs the benefits of local control [32]. This study marks a significant advance in our understanding of the potential mechanistic synergy of combining hypofractionated RT and immunotherapy to treat primary refractory DLBCL. Accumulating evidence indicates that the residual tumor (defined as refractory) burden is a highly significant prognostic factor, irrespective of whether systemic or localized therapies are used [33, 34, 35, 36]. Given the low risk of widespread failure, the benefit of adding RT to systemic therapy might be greater in patients with a low residual tumor burden. Our previous study confirmed that the presence of a single residual lesion is associated with a higher PFS rate [20]. In refractory DLBCL, R‐GemOx‐based regimens have demonstrated modest efficacy, with reported CR rates of 25.3–29% and ORRs of 40.7–45% across multicenter trials (the STARGL and LEO CReWE studies) [37, 38]. While the addition of immunotherapy (e.g., atezolizumab) to salvage chemoimmunotherapy improved outcomes (CR 33%) [39]. These results remain inferior to the CR rate of 61.5% observed in our study using hypofractionated RT combined with R‐GemOx–zimberelimab. These findings are consistent with previous observations, indicating that patients with oligometastatic or oligoresidual disease might derive greater benefit from RT. This stark contrast underscores the potential advantage of leveraging localized RT to overcome systemic therapy resistance in refractory DLBCL. Additionally, the toxicity of hypofractionated RT was manageable, mainly because of the use of PET‐guided Involved Site Radiation Therapy [21, 32]. Importantly, no grade 4 or 5 TRAEs occurred during the immunochemotherapy phase, which might be partly attributed to the use of zimberelimab, a newly developed fully human anti‐PD‐1 monoclonal antibody. This combined treatment approach represents one of the key innovations of our study. While our research primarily focused on the combination of hypofractionated RT and ICIs, several emerging therapies, including polatuzumab vedotin, glofitamab, and CAR‐T cell therapy, have shown promise as effective salvage strategies in refractory DLBCL [10, 40, 41] Notably, the treatment approach explored in our study aligns with the current strategy of combining RT with CAR‐T cell therapy, which has shown synergistic potential in overcoming resistance and improving outcomes. Although the combination of RT with glofitamab and polatuzumab vedotin has been rarely reported [42], it holds significant promise and warrants further exploration. This approach could offer a new avenue to enhance the immune response in refractory DLBCL, potentially improving both local control and systemic efficacy.

In oligometastatic or oligoresidual disease, combining ICIs with hypofractionated RT has shown synergistic antitumor effects in several epithelial cancers, including lung, esophagogastric, and kidney cancers [43, 44, 45, 46]. However, hypofractionated RT has long been neglected in lymphoma treatment because of this cancer's high sensitivity to radiation [47]. With the advent of CAR‐T cell therapy, hypofractionated RT has quickly become a recommended regimen [13, 14]. Notably, combining hypofractionated RT with CAR‐T cell therapy has demonstrated synergistic potential: preclinical observations indicated that RT enhances the infiltration and activity of CD3+ and CD8+ T cells within the TME, while also upregulating cytotoxic CD107+ T cells, a marker of enhanced degranulation and tumor cell killing [48]. Therefore, Reducing the tumor burden and potentially increasing tumor immunogenicity with hypofractionated RT are believed to be two key factors to improve CAR‐T cell therapy [15, 16]. Inspired by this strategy, we propose that combining hypofractionated RT with ICIs could represent a new treatment method for DLBCL. ICIs harness T cells to eradicate tumor cells, and accumulating evidence indicates that the TIME is strongly associated with the efficacy of immunotherapies [49]. Our DSP data showed similar results, thereby facilitating the investigation of compartment‐specific expression without tissue manipulation. Additionally, CellChat analysis revealed that CCR7 was the most important receptor, which is a hallmark of central memory and naive T cells, regulating their homing to lymph nodes. The relationship between clinical outcomes and the transcriptome results was verified using mIHC. Our analysis revealed that biomarkers found in specific compartments differed in their expression between lymphoma and T cells, indicating an interaction between the two regions.

For patients with DLBCL treated with R‐CHOP, distinct molecular subtypes might benefit from specific novel targeted agents, which has been a cornerstone of certain clinical trials [50, 51]. Mutations in epigenetic factors, including lysine methyltransferase 2D, enhancer of zeste 2 polycomb repressive complex 2 subunit, DNA methyltransferase 3 alpha, and CREB binding protein, have been well documented to alter the composition of immune cells in the TIME and might represent key therapeutic targets [52, 53]. However, studying immune cells in lymphoma is challenging because the disease originates from lymphocytes [54]. In double‐expressor DLBCL, R‐CHOP combined with chidamide (a histone deacetylase inhibitor) improved patient survival in a recent phase 3 clinical trial [55]. This observation is particularly noteworthy because ICIs are remarkably ineffective in DLBCL [56], potentially because epigenetic factors recruit various immunosuppressive cells by secreting cytokines. Despite the potential advantages of combining epidrugs with ICIs, a clear clinical benefit has not been demonstrated in lymphomas, except for natural killer/T cell lymphoma [57]. Our data suggest that patients with primary refractory DLBCL achieved significant remission of residual lesions using hypofractionated RT, which could enhance the efficacy of ICIs by reshaping the suppressive TIME, with the increased T cell infiltration induced by RT being one of the potential key mechanisms. The synergy of epidrugs, ICIs, and hypofractionated RT deserves further exploration.

Our study has some limitations. First, as a preliminary analysis for a phase 2 study, it involved a relatively small sample size and a brief follow‐up period. Despite these constraints, the initial results are sufficiently encouraging to justify further enrollment. Second, the single‐arm design of this study precludes a definitive comparison with standard‐of‐care therapies. Although ASCT and CAR‐T cell therapy are the standard salvage treatments, their implementation is often hindered by advanced patient age and prohibitive costs. Consequently, RT, either alone or combined with immunotherapy, has emerged as a viable option for patients at low risk of widespread failure, offering high efficacy and low toxicity. Third, our focus was limited to the molecular features of lymphoma and tumor‐infiltrating T cells. It has been challenging to collect tissue samples at multiple time points during treatment because of the location of the tumors in the abdominal and thoracic cavities, thereby hindering dynamic assessments of changes in the TME. In the next phase of our research, we plan to employ animal models to facilitate the collection of multiple tissue samples during the course of treatment. Furthermore, to enhance the depth of our analysis for selected patients, we aim to incorporate single‐cell RNA sequencing and spatial transcriptomics to better characterize the interactions between tumor cells and the surrounding stromal cells. Last, the therapeutic benefit for patients with acquired refractory disease was not evaluated in this study. Future research should explore whether hypofractionated RT and ICIs can benefit a broader patient population.

## 4 Conclusions

This study provides strong evidence that hypofractionated RT combined with zimberelimab is both effective and safe to treat primary refractory diffuse DLBCL. Our detailed compartment‐specific transcriptomic and immune analysis offers new insights to better select primary refractory patients for this novel treatment strategy. We are confident that the study will continue until the enrollment goal is achieved.

## 5 Materials and Methods

### 5.1 Study Design and Participants

This phase II, open‐label, single‐arm multicenter study (ChiCTR2200060059) aimed to evaluate the efficacy, safety, and biomarkers of sequential hypofractionated RT and immuno‐chemotherapy in patients with primary refractory DLBCL. The institutional review board of Fujian Medical University Union Hospital approved the protocol on March 31, 2022 (approval number 2022YF019‐01). The study adhered to the World Medical Association Declaration of Helsinki principles. Participants gave written informed consent prior to inclusion in the study.

Patients had to have histologically confirmed DLBCL and not have achieved a CR after four to six cycles of standard first‐line therapy (R‐CHOP or R‐CHOP‐like regimens) to be eligible for inclusion. Additional inclusion criteria included an ECOG performance status of 0–2, age between 18 and 75 years, and an expected survival of at least 12 weeks. Key exclusion criteria were the presence of primary mediastinal, testicular, cutaneous, or central nervous system lymphoma; involvement of the central nervous system; a prior history of severe autoimmune disease; previous treatment with an ICI; and unwillingness or inability to undergo PET/CT examinations.

### 5.2 Procedures

The CONSORT diagram is presented in Figure 1. Baseline evaluation included a complete medical history, general examination, PET/CT, and hematological and biochemical assessments. All enrolled patients received five 21‐day cycles of the ICI zimberelimab (240 mg/dose, administered intravenously) combined with four 21‐day cycles of the R‐GemOx regimen (rituximab 375 mg/m^2^ intravenously on day 1, gemcitabine 1000 mg/m^2^ intravenously on days 1 and 8, and oxaliplatin 100 mg/m^2^ intravenously on day 1). The first dose of zimberelimab was administered before RT, as illustrated in Figure 1A.

The target volumes and radiation doses were defined based on the International Lymphoma Radiation Oncology Group (ILROG) guidelines [21, 32]. The gross tumor volume (GTV) was delineated as the hypermetabolic residual lesions identified by PET/CT imaging, with diagnostic criteria based on the Deauville 5‐point scale, where scores of 4 and 5 are considered positive. The CTV encompassed the GTV along with adjacent regions initially involved at diagnosis that had responded to first‐line chemotherapy. Both the GTV and CTV were expanded to create the planning gross target volume (PGTV) and the PTV using a 3–7 mm margin. Hypofractionated RT, with an integrated boost, was administered in 12 fractions, delivering a total dose of 36 Gy to the PGTV and 24 Gy to the PTV (Figure S2) [20].

### 5.3 Assessment

The primary endpoint was the CR rate, defined as the proportion of patients achieving a CR after treatment, as assessed by investigators using the Lugano 2014 criteria [58]. Secondary endpoints included safety, OS, PFS, and the identification of biomarkers predictive of treatment efficacy. In‐field and out‐of‐field progression for RT were defined according to our previous study [20]. Toxicities associated with immuno‐chemotherapy and RT were documented according to the National Cancer Institute Common Terminology Criteria for Adverse Events (version 4). This study offers early insights into treatment efficacy and the rate and severity of adverse events. The OS was measured from the time of study enrollment until death from any cause, whereas PFS was measured from enrollment to either progression or death from any cause [59].

### 5.4 Digital Spatial Profiling

To comprehensively explore differences in the tumor ecosystem at the gene level, we utilized DSP with the Nanostring GeoMx human whole‐transcriptome atlas (Nanostring, Seattle, WA, USA), which covers over 18,000 genes. Five‐micron formalin‐fixed/paraffin‐embedded (FFPE) tissue samples, obtained before treatment, were processed according to the GeoMx DSP protocols. The slides were stained with fluorescently labeled morphology markers: Syto13 (nucleus; NanoString, 121300303), CD20 (also known as membrane spanning 4‐domains A1) (lymphoma; EP459Y clone; Abcam, Cambridge, MA, USA), and CD3 (T cells; SP162 clone; Abcam) (Figure 1C). Multiple polygonal‐shaped ROIs were selected as representative parts of the lymphoma and immune areas, as defined morphologically by a pathologist (X.‐M. Hu). Custom areas of illumination (AOIs) for lymphoma and T cells within an ROI were segmented based on CD20 (+) and CD3 (+) markers. A total of 45 AOIs were profiled separately.

For the segmented AOIs, a dual digital mirror device module was utilized to release ligated index oligonucleotides. These oligonucleotides were then collected by a microcapillary arm and aspirated into individual wells of a microtiter plate. Following the processing of all ROIs and the collection of barcoded oligonucleotides, digital counting was carried out using the nCounter system, and the data were subsequently processed. Libraries were prepared following NanoString's next‐generation sequencing (NGS) readout protocol. FASTQ files generated by Illumina sequencing (Illumina Inc., San Diego, CA, USA) were processed into digital count conversion (DCC) files using the GeoMx NGS Pipeline Software (V2.2). The R package GeomxTools (V3.0.1)(https://www.R‐project.org/) was used to analyze the DCC files. The ROIs with a read alignment rate below 80% relative to the template sequence were excluded based on technical signal quality control. Three indicators were used to assess the technical background: The no template control (NTC) count, the negative probe count, and the ROI parameters. ROIs with NTCs exceeding 36,000 were eliminated, and the threshold for negative probe counts was set at four counts. Additionally, parameters such as nuclei counts (greater than 90) and surface area (greater than 4000 square microns) were used to qualify ROIs.

### 5.5 Ligand and Receptor‐Based Cell Interaction

To investigate how CD20(+) lymphomas influence neighboring CD3(+) T cells in non‐CR patients, these were designated as “sender cells” and “receiver cells,” respectively. ​In the context of ligand and receptor interactions, DEGs that were upregulated in lymphomas were considered as “senders,” while those in T cells were considered “receivers.” Potential ligands and receptors were identified through the CellChatDB (V2.1.0) computational framework for ligand–receptor interactions [60]. NicheNet analysis was employed to determine potential interactions between the identified ligands and receptors [61].

### 5.6 Fluorescent mIHC

To further validate the association between treatment efficacy and T cell infiltration, ligand–receptor interactions identified at the DSP level were assessed using an mIHC assay. Consecutive sections from the same FFPE samples used for DSP analysis were employed. Following deparaffinization in xylene for 30 min, slides were rehydrated in absolute ethyl alcohol with two 5‐min washes, 95% ethyl alcohol (for 5 min), and 75% ethyl alcohol (for 2 min). The slides were then washed three times with distilled water. For heat‐induced epitope retrieval, the slides underwent heat treatment in boiling EDTA buffer (ZLI‐9079; ZSbio, Beijing, China) for 15 min using a microwave oven. Blocking was carried out using Antibody Diluent/Block from Alpha X Bio (Beijing, China). The AlphaXPainter X30 system (Alpha X Bio) was used for the mIHC experiments, following the protocol provided by the manufacturer. The panel of primary antibodies used comprised: anti‐CD4 molecule (CD4) (Cat# ZM0418, diluted 1:200; ZSGB‐Bio, Beijing, China), anti‐CD8 molecule (CD8) (Cat# ZA0508, diluted 1:200; ZSGB‐Bio), anti‐CD20 (Cat# ab78237, diluted 1:200; Abcam), anti‐CD45 long isoform (CD45RA; Cat# ZM0053, diluted working solution; ZSGB‐Bio), anti‐CD45 short isoform (CD45RO; Cat# ZM0055, diluted 1:200; ZSGB‐Bio), and anti‐C–C motif chemokine receptor 7 (CCR7; Cat# aab253187, diluted 1:300; Abcam). Nuclear staining was performed using 4′,6‐diamidino‐2‐phenylindole (DAPI) (Alpha X Bio).

Slides were treated with primary antibodies and incubated at 37°C for 1 h. Thereafter, the slides were incubated with Alpha X Polymer HRP Ms+Rb (horseradish peroxidase‐conjugated, recognizing mouse and rabbit antibodies; Alpha X Bio) for 10 min at 37°C. Visualization was carried out using the Alpha X 7‐Color IHC Kit (AXT37100031; Alpha X Bio). To eliminate all primary and secondary antibodies, heat‐induced epitope retrieval was conducted again after each staining cycle. The slides were counterstained with DAPI for 5 min and then mounted in Antifade Mounting Medium (I0052; NobleRyder, Beijing, China). The slides were scanned using a ZEISS AXIOSCAN 7 scanner (ZEISS, Oberkochen, Germany), and the images were analyzed using HALO software (v3.6, Indica Labs, Albuquerque, NM, USA).

### 5.7 Statistical Analyses

The study employed a Simon's optimal two‐stage design, with a power of 80% and a one‐sided α of 0.05, to exclude a CR rate of 20% or less (null hypothesis) when the target CR rate was set at 40% (Figure 1B). In the first stage, if three or more patients achieved a CR among the initial 13 patients, an additional 30 patients would be recruited. Accounting for an estimated 20% dropout rate, the total required sample size was calculated as 54 patients. The CR rate and its 95% confidence interval (CI) were calculated using the Clopper–Pearson method. The chi‐squared or Fisher's exact test were applied for group comparisons. The OS and PFS analyses were conducted using the Kaplan–Meier method, and survival curves were assessed with the log‐rank test. The demographic characteristics, safety profiles, and dosimetric parameters were summarized using descriptive statistics.

Differential gene expression analysis was conducted using edgeR (v3.38.4) with the following criteria: a FDR of *p* < 0.05 and an absolute expression fold change > 1.0. The Benjamini–Hochberg method was used to adjust the *p* values for multiple comparisons. Volcano plots and heatmaps displaying DEGs were generated using the R ggplot package (v3.4.4). We explored pathway enrichment using the Reactome database [62]. Differentially overexpressed ligands and receptors between lymphoma and T cells were identified using NicheNet analyses (version 2.1.0). Immune cell densities were assessed by counting positively stained cells per mm^2^ across the total area. Differences in tumor‐infiltrating immune cell densities between the CR and non‐CR groups were compared using Student's t‐test. Additionally, prognostic analysis based on pretreatment cell proportions was performed using the R xCell package (v1.1.0) from the DLBCL GSE31312 dataset. Based on the cell abundance thresholds identified using the R survival ROC package (v 1.0.3.1), survival curves were generated using the R survival package (v3.8) to assess the impact of immune cell infiltration on patient survival. All computational analyses were carried out in the R statistical environment (v4.2.1).

## Author Contributions

*Conception and design*: Yong Yang, Yu Jing, Hai‐Ying Fu, and Ting‐Bo Liu. *Financial support*: Yong Yang, Ting‐Bo Liu, and Hai‐Ying Fu. *Administrative support*: Yong Yang and Ting‐Bo Liu. *Provision of study material or patients*: all authors. *Collection and assembly of data*: Yong Yang, Xiao‐Mei Hu, Si‐Lin Chen, Rui‐Zhi Zhao, Cheng Huang, and Jiang‐Rui Guo. *Data analysis and interpretation*: Yong Yang, Xiao‐Mei Hu, Cheng Huang, Rui‐Zhi Zhao, and Si‐Lin Chen. *Manuscript writing*: all authors. *Final approval of manuscript*: all authors. *Accountable for all aspects of the work*: all authors. All authors have read and approved the final manuscript.

## Ethics Statement

This study was approved by the Institutional Review Board of Fujian Medical University Union Hospital, and registered with chictr.org.cn, ChiCTR2200060059.

## Consent

All participants provided written informed consent before treatment, and this trial adhered to all relevant ethical considerations.

## Conflicts of Interest

The authors declare no conflicts of interest.

## Supporting information



Supporting Information

## Data Availability

The sequencing data supporting the findings of this study have been deposited in the China National Center for Bioinformation (CNCB) OMIX database (https://www.cncb.ac.cn/?lang=en) under accession number OMIX006705. All other data supporting the findings of this study are available from the corresponding author, Dr Yong Yang (dr_yangyong1983@163.com), upon reasonable request.
