# Supplementary material for: Phase II Trial of Hypofractionated Radiotherapy and Immunochemotherapy in Primary Refractory Diffuse Large B‐Cell Lymphoma: Preliminary Results and Insights from Digital Spatial Profiling
Source: MedComm (2020). 2025 May 25;6(6):e70225. doi: 10.1002/mco2.70225 (PMC12103654; doi:10.1002/mco2.70225)
Supplement: Supplementary file 1 — Supporting Information [file MCO2-6-e70225-s001.docx]

**Supplementary Information**

**Title:**
Phase II Trial of Hypofractionated Radiotherapy and Immunochemotherapy in Primary Refractory Diffuse Large B-cell Lymphoma: Preliminary Results and Insights from Digital Spatial Profiling

**Contents of Supplementary Information**

- **Supplemental Appendix Table 1.**
  Predicted Ligand-Receptor Interactions Based on Differentially Expressed Genes (DEGs) in CD20-Rich and CD3-Rich Areas of Interest (AOIs)
- **Supplemental Appendix Figure 1.**
  Association Between T Cell Abundance and Progression-Free Survival (PFS) in DLBCL Patients
- **Supplemental Appendix Figure 2.**
  Radiotherapy treatment planning for hypofractionated RT with an integrated boost

| **Gene name** | **Gene id** | **log2FC** | **P-Value** | **FDR** | **as.ligand.in.list** | **as.receptor.in.list** | **partner.in.list** |
| --- | --- | --- | --- | --- | --- | --- | --- |
| C3 | ENSG00000125730 | -1.067104609 | 0.001476373 | 0.021142167 | TRUE |  | ITGB2,ITGAX |
| CCL19 | ENSG00000172724 | -2.902026337 | 2.06E-05 | 0.00189121 | TRUE |  | CCR7 |
| CCL21 | ENSG00000137077 | -3.477931831 | 0.000347944 | 0.009098867 | TRUE |  | CCR7 |
| CCR7 | ENSG00000126353 | -1.304846444 | 7.17E-05 | 0.003968075 |  | TRUE | CCL19,CCL21 |
| CD22 | ENSG00000012124 | -1.213093369 | 0.000763671 | 0.014439993 | TRUE | TRUE | CCL19,CCL21 |
| CD69 | ENSG00000110848 | -1.194726929 | 0.000539139 | 0.011697722 | TRUE |  |  |
| IL7R | ENSG00000168685 | -1.499889817 | 2.05E-05 | 0.00189121 |  | TRUE |  |
| ITGAX | ENSG00000140678 | -1.084437906 | 4.86E-05 | 0.003116667 |  | TRUE | C3 |
| ITGB2 | ENSG00000160255 | -1.185828917 | 0.001795247 | 0.023315583 |  | TRUE | C3,C3 |
| SELL | ENSG00000188404 | -1.213316663 | 0.000606663 | 0.01251828 | TRUE | TRUE | CCL19,CCL21 |
| SELPLG | ENSG00000110876 | -1.033289481 | 0.000411328 | 0.009819556 | TRUE |  | SELL |

**Supplemental appendix Tabel 1.** Predicted Ligand-Receptor Interactions Based on Differentially Expressed Genes (DEGs) in CD20-Rich and CD3-Rich Areas of Interest (AOIs)


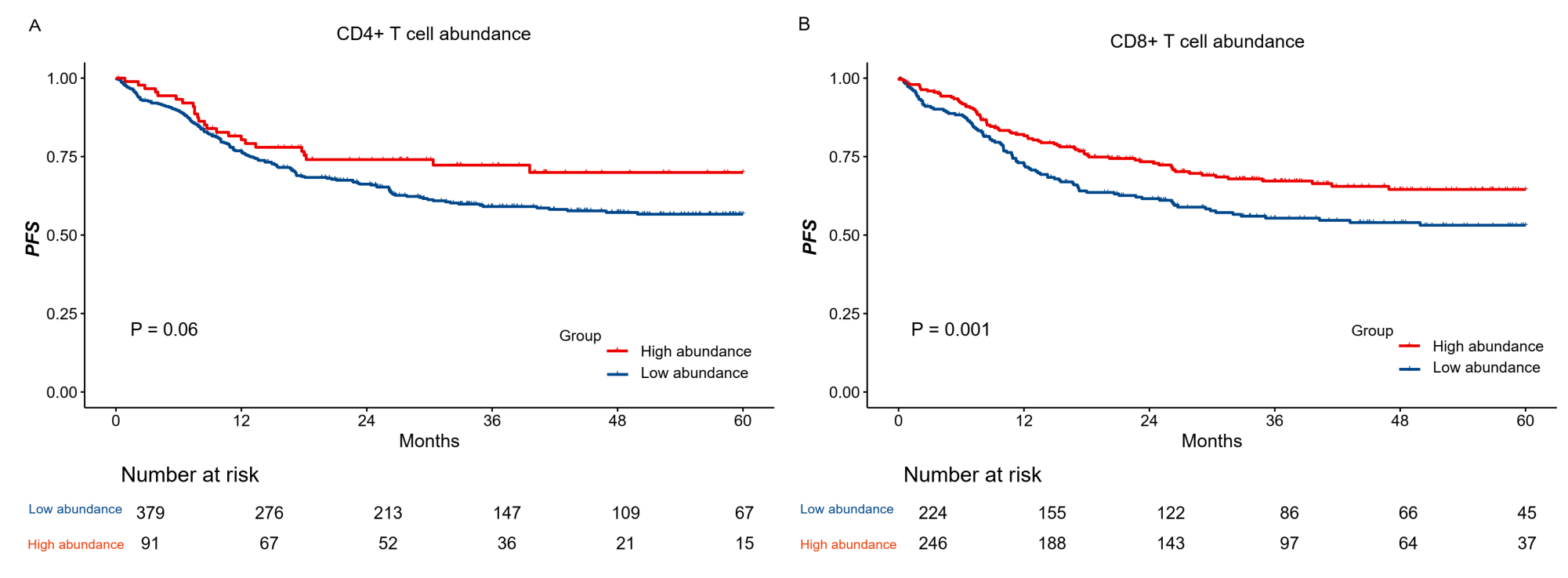


**Supplemental appendix Figure 1.** Association Between T Cell Abundance and Progression-Free Survival (PFS) in DLBCL Patients.

A, CD4+ T Cell Abundance and PFS: Kaplan–Meier survival curve illustrating the association between CD4+ T cell abundance and PFS in DLBCL patients. B, CD8+ T Cell Abundance and PFS: Kaplan–Meier survival curve showing the relationship between CD8+ T cell abundance and PFS. The x-axis represents time in months, while the y-axis represents the probability of PFS. The number of patients at risk at each time point is displayed below the plot.

**
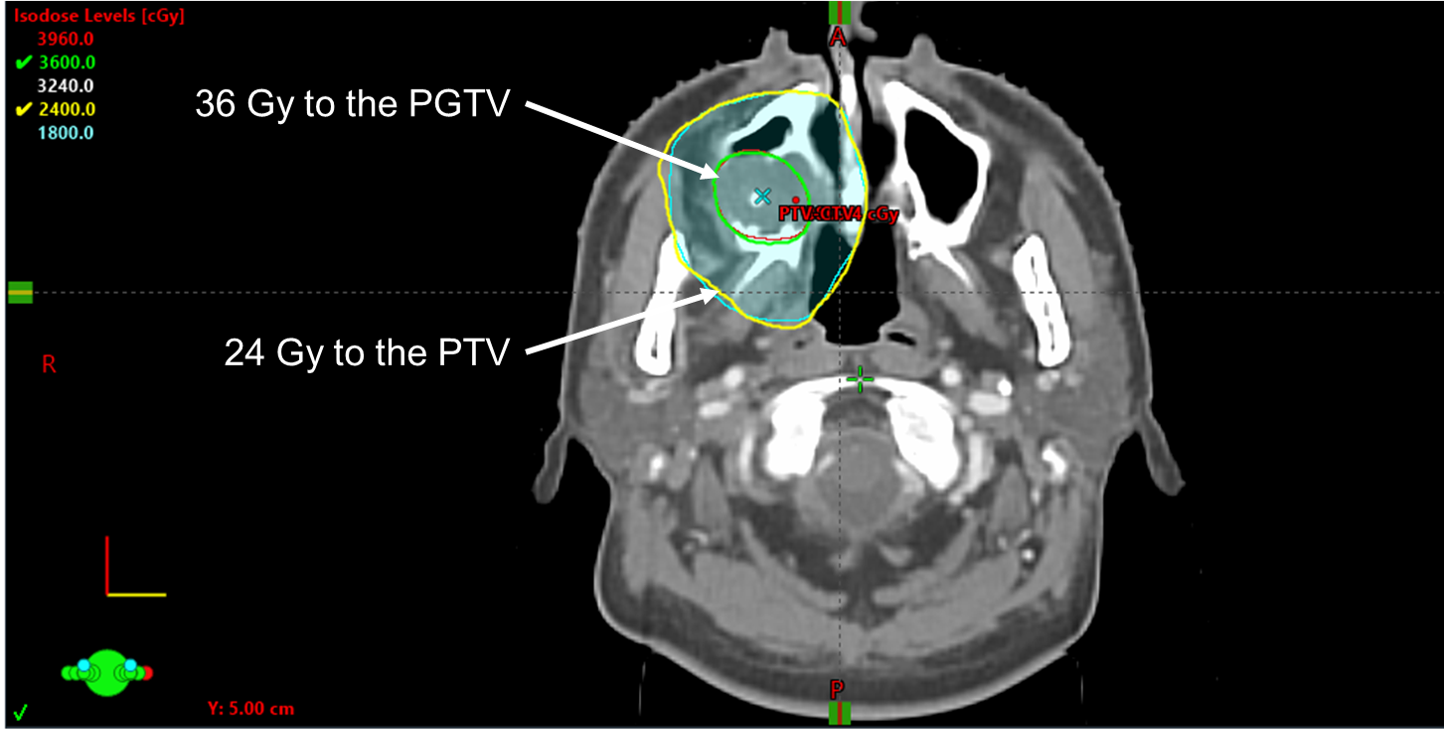
**

**Supplemental appendix Figure 2.** Radiotherapy treatment planning for hypofractionated RT with an integrated boost

Representative treatment planning system (TPS) screenshot illustrating the dose distribution for hypofractionated radiotherapy (RT). The planning gross target volume (PGTV) received a total dose of 36 Gy (yellow contour) in 12 fraction, while the planning target volume (PTV) received 24 Gy (green contour) in 12 fractions. The isodose lines represent different radiation dose levels, ensuring precise dose delivery to the target regions while sparing surrounding normal tissues.
